# Parkinson's disease progression is shaped by longitudinal changes in cerebral compensation

**DOI:** 10.1093/brain/awaf302

**Published:** 2025-08-12

**Authors:** Martin E Johansson, Ivan Toni, Bastiaan R Bloem, Rick C Helmich

**Affiliations:** Radboud University Medical Center, Donders Institute for Brain, Cognition and Behaviour, Centre of Expertise for Parkinson & Movement Disorders, Nijmegen 6500 HB, The Netherlands; Radboud University, Donders Institute for Brain, Cognition and Behaviour, Nijmegen 6525 EN, The Netherlands; Radboud University Medical Center, Donders Institute for Brain, Cognition and Behaviour, Centre of Expertise for Parkinson & Movement Disorders, Nijmegen 6500 HB, The Netherlands; Radboud University Medical Center, Donders Institute for Brain, Cognition and Behaviour, Centre of Expertise for Parkinson & Movement Disorders, Nijmegen 6500 HB, The Netherlands

**Keywords:** Parkinson’s disease, action selection, functional magnetic resonance imaging, compensation, disease progression, longitudinal

## Abstract

Parkinson's disease is a common and debilitating neurodegenerative disorder characterized by motor slowing (bradykinesia), which is thought to arise mainly owing to nigrostriatal dopaminergic cell loss. Paradoxically, longitudinal changes in striatal dopamine are poorly related to the progression of bradykinesia, indicating that other pathophysiological mechanisms play a role. In line with this, cross-sectional studies have shown that more benign motor phenotypes of Parkinson's disease are characterized by increased activity in the parieto-premotor cortex, indicative of cerebral compensation. However, the role of cerebral compensation in disease progression remains unclear. Here, we used a longitudinal design to test the hypothesis that the clinical progression of bradykinesia in Parkinson's disease is related to a decline in compensatory parieto-premotor function, over and above worsening nigrostriatal cell loss.

We used a validated action selection task in combination with functional MRI to measure motor- and selection-related brain activity relative to the most-affected hand in a large sample of 351 patients with Parkinson's disease (≤5 years disease duration) and 60 healthy control subjects. In addition, we used diffusion-weighted MRI to obtain structural indices of substantia nigra and cerebral cortex integrity. These measurements were acquired at baseline and at 2-year follow-up, enabling us to compare longitudinal changes in brain metrics between patients and controls and to investigate their relationships with clinical metrics of bradykinesia progression.

Consistent with our hypothesis, we observed that bradykinesia progression was inversely related to longitudinal changes in selection-related dorsal premotor cortex activity, suggesting that faster loss of cortical compensation contributes to faster symptom worsening. Importantly, this relationship remained after adjusting for longitudinal changes in the functional and structural integrity of the nigrostriatal system, indicating that bradykinesia progression is determined uniquely by loss of cortical compensation. In group comparisons of longitudinal change, patients with Parkinson's disease showed an overall reduction in putamen activity, which did not decrease further over time, in combination with an acceleration of structural decline in the substantia nigra and the premotor cortex. Despite showing expected patterns of Parkinson's disease pathology, neither of these metrics was correlated with bradykinesia progression.

We conclude that the progression of bradykinesia in Parkinson's disease is determined by longitudinal changes in compensatory premotor cortex function. This presents opportunities to develop new progression-slowing interventions that focus on preserving and enhancing cortical compensation.

## Teaser

Johansson *et al.* leverage longitudinal clinical and task-based fMRI data to demonstrate that the progression of bradykinesia in Parkinson's disease relates more strongly to changes in compensatory mechanisms in the dorsal premotor cortex, than to advancing nigrostriatal dysfunction.

## Introduction

Parkinson's disease (PD) is an increasingly common neurodegenerative disorder characterized, among other symptoms, by progressive motor slowing (bradykinesia), which gradually impairs the ability to engage in everyday activities.^1-3^ Disease-modifying treatments are urgently needed, but their development is currently hindered by a lack of biomarkers that can adequately monitor the pathophysiological mechanisms that drive the progression of symptoms.^4,5^ Converging evidence now indicates that the clinical progression of PD is partly determined by changes in compensatory hyperactivity in the cerebral cortex,^6-9^ and not only nigrostriatal dysfunction (the pathophysiological hallmark of PD), which opens possibilities to develop new biomarkers that can be leveraged to advance treatment development. Here, we exploit a unique 2-year longitudinal dataset to test the hypothesis that bradykinesia progression relates more strongly to declining cortical compensation than to loss of nigrostriatal function.

The motor symptoms of PD, and bradykinesia in particular, have traditionally been attributed to loss of dopaminergic cells in the substantia nigra and depletion of dopamine in the striatum, particularly in motor territories of the posterior putamen. In turn, striatal dopamine depletion leads to excessive inhibitory output from basal ganglia nuclei, which limits activation of corticostriatal pathways that encode movement vigour.^10-13^ Despite providing a compelling explanation for the presence of bradykinesia, nigrostriatal cell loss might play only a limited role in determining the clinical progression of PD; striatal dopamine depletion begins several years before the onset of bradykinesia,^14,15^ is largely complete in the posterior putamen within 4 years after diagnosis,^16^ and is generally a poor predictor of symptom worsening.^17-22^ Recent data show that only progression of motor symptoms on the least-affected side, but not the most-affected side, has a significant (albeit weak) correlation with striatal dopamine depletion.^23^ These observations strongly imply that the progression of bradykinesia must be shaped by additional pathophysiological mechanisms.

PD-related deficits in the nigrostriatal system develop slowly and are continuously offset by compensatory adaptations in brain function,^24-28^ particularly involving areas that remain relatively spared from neurodegeneration during early disease stages,^8,9,29^ potentially explaining why motor symptoms appear only after ∼60% of dopamine is lost from the posterior putamen.^30^ As nigrostriatal dysfunction worsens and neurodegeneration spreads throughout the brain, compensatory adaptations gradually fail to preserve motor function, and symptoms worsen.^27^ Compensatory decline therefore constitutes a promising index of clinical progression and a potential target for disease-modifying interventions.

**Figure 1:**
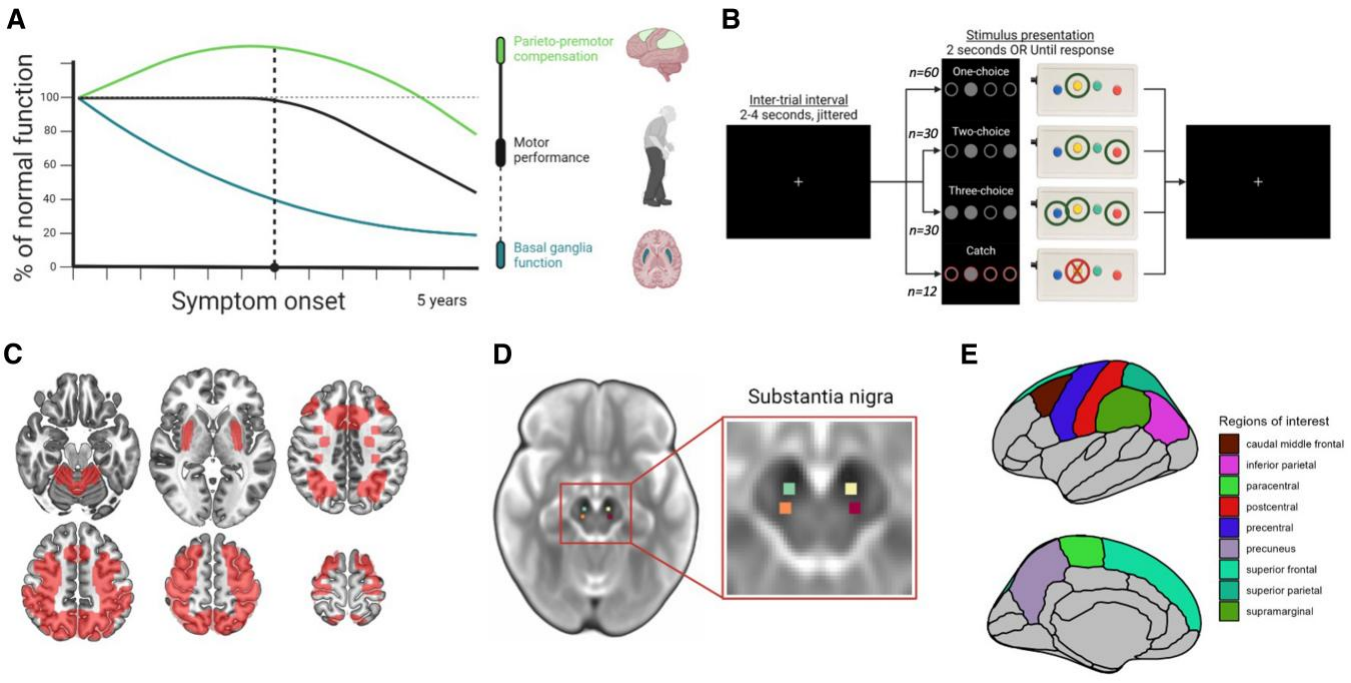
**Study design.** (**A**) Hypothetical model of Parkinson’s disease progression. Loss of compensation is predicted to have a stronger influence on clinical progression than increasing basal ganglia dysfunction. Reproduced with permission from Johansson *et al*.^7^ (**B**) Action selection task. (**C**) Mask used to constrain voxel-wise group and brain–clinical correlation analyses to regions implicated in cortical compensation and basal ganglia dysfunction based on previous findings.^7^ (**D**) Masks of substantia nigra subregions manually drawn on a study-specific b0-template and used to extract free water values. (**E**) Regions in the Desikan–Killiany atlas used to extract surface-projected mean diffusivity.

Recent evidence suggests that compensatory decline can be measured using functional MRI (fMRI).^5,7,31^ FMRI studies have consistently shown that PD is characterized by movement-related hyperactivity in parietal and premotor regions of the cerebral cortex, which might be compensatory in nature.^8,9^ In support of this conjecture, we recently demonstrated that parieto-premotor hyperactivity, measured with fMRI in combination with a visuomotor task designed to elicit PD-related compensation, is inversely related to the severity of bradykinesia.^7^ We found no relationship between the severity of bradykinesia and basal ganglia activity, which was consistently reduced in individuals with PD in comparison to healthy controls. These cross-sectional findings indicate that parieto-premotor hyperactivity helps to maintain motor performance by compensating for reduced basal ganglia activity, providing initial support for the hypothesis that motor progression is more closely linked to declining cortical compensation than to increasing nigrostriatal dysfunction (Fig. 1A). This hypothesis is supported further by findings from an intervention trial demonstrating that aerobic exercise attenuates the clinical progression of PD by enhancing cortical compensation.^6^

Here, we combine longitudinal task-based fMRI and clinical assessments to investigate the neural mechanisms underlying individual differences in 2-year clinical PD progression, focusing particularly on the role of longitudinal changes in cortical compensation. Our results indicate that the progression rates of bradykinesia are specifically shaped by loss of cortical compensation, rather than by increasing nigrostriatal dysfunction.

## Materials and methods

### Participants

Three hundred and sixty-seven patients diagnosed with idiopathic PD and 60 healthy controls underwent baseline MRI and clinical assessments. Three hundred and thirty-eight patients and 56 healthy controls returned at 2-year follow-up. Ten patients were re-diagnosed at baseline (two parkinsonism and eight other), and six patients were re-diagnosed at 2-year follow-up (three multiple-system atrophy, two progressive supranuclear palsy and one other). The exclusion of these patients led to a total sample size of 351 (baseline) and 329 (follow-up) patients. Detailed numbers of exclusions in analyses of clinical measurements, task performance and MRI data can be found in the Supplementary material. All data were retrieved from the Personalized Parkinson Project (ClinicalTrials.gov identifiers: NCT03364894 and NCT05169827) database in January 2024. Written informed consent was obtained for all participants in accordance with the Declaration of Helsinki. The study was approved by a medical ethical committee (METC Oost-Nederland, formerly CMO Arnhem-Nijmegen; #2016-2934 and #2018-4785). Further details about the measurement sessions are provided in the protocol of the Personalized Parkinson Project.^32^ Demographic information can be found in Table 1. More detailed information about data acquisition and procedures for the estimation of task-based brain activity can be found in a previous publication.^7^

**Table 1:** Baseline characteristics and demographic information

Measurement	Control	Patient	*P*-value
*n*, baseline/follow-up	60/56	351/328	–
Age, years	60.0 (9.6)	62.7 (8.5)	0.046
Sex, female/male	27/33	129/222	0.283
Years of education	16.2 (3.3)	17.2 (4.1)	0.041
Time to follow-up, years	2.2 (0.1)	2.4 (0.3)	<0.001
Dominant hand, left/right/none	55/5/0	295/49/7	0.249
Responding hand, left/right	29/31	163/180	1.000
MoCA	27.6 (1.9)	26.7 (2.6)	0.004
Most affected side, left/right/none	–	163/173/17	–
Disease duration, years	–	2.9 (1.4)	–
Hoehn and Yahr stage	–	41/264/43/3/0	–
Medication use, no/yes	–	17/334	–
LEDD, mg	–	548.3 (319.8)	–
Fluctuations (functional impact)^a^	–	224/54/25/37/10	–
Fluctuations (complexity)^b^	–	194/89/35/3/18	–
**MDS-UPDRS III: OFF**			
Total	–	33.5 (13.0)	–
Bradykinesia	–	16.6 (7.4)	–
**MDS-UPDRS III: ON**			
Total	–	28.6 (12.5)	–
Bradykinesia	–	14.3 (7.2)	–

LEDD = Levodopa Equivalent Dosage; MDS-UPDRS = Movement Disorder Society Unified Parkinson’s Disease Rating Scale; MoCA = Montreal Cognitive Assessment. *P*-values reflect outcomes of independent-samples *t*-tests.

^a^MDS-UPDRS IV item 4.4.

^b^MDS-UPDRS IV item 4.5.

### Clinical measurements

Motor symptoms were clinically assessed with part III of the Movement Disorders Society-sponsored Revision of the Unified Parkinson's Disease Rating Scale (MDS-UPDRS III)^33^ in OFF-medicated (morning; <12 h withdrawal) and ON-medicated states (afternoon; ∼1 h after intake, at a time of subjective optimal improvement). Severity of bradykinesia was defined as the summed scores of 11 items from the MDS-UPDRS part III (4–9 and 14).^34^ Bradykinesia progression was defined as the between-session delta (Δ; 2-year follow-up minus baseline) and was calculated for both ON- and OFF-state scores. ON-state progression was used as the primary outcome in all correlation analyses. Although suboptimal as a marker of ‘pure’ disease progression, ON-state progression more closely reflects the state of patients during scanning, which was done in the ON-medicated state. Moreover, ON- and OFF-state progression were correlated substantially (see later). Furthermore, dopaminergic medication might boost compensatory mechanisms that rely on the relatively spared anterior striatum.^25^ Bradykinesia progression scores above or below 3 SD from the mean were deemed unreliable and excluded from correlation analyses (only one patient was excluded following this criterion). Cognitive symptoms were assessed using the Montreal Cognitive Assessment (MoCA),^35^ and medication dosages were quantified as the levodopa-equivalent daily dosage (LEDD). Motor fluctuations were assessed using items 4.4 (functional impact) and 4.5 (complexity) of the MDS-UPDRS part IV scale. Additionally, motor symptom asymmetry was computed as the difference between left- and right-sided bradykinetic-rigid items of the MDS-UPDRS III.^34^ Progression on these metrics was calculated in the same way as for bradykinesia. Missing clinical scores were imputed through predictive mean matching.^36^

### Action selection task

The behavioural task used in this study (Fig. 1B) and the metrics that were derived from it have been described in detail elsewhere.^7^ In short, participants were instructed to respond to a single highlighted cue with a button press as quickly and as accurately as possible, and to make equal use of all available response options. The number of highlighted cues varied from one (single-choice) to two and three (multiple-choice). Selecting between multiple, equally relevant response options introduces an element of mild cognitive effort that overloads the dysfunctional basal ganglia motor network in PD, thereby eliciting recruitment of compensatory resources in brain areas that remain relatively spared from pathology.^25^ The primary behavioural metric derived from the task was response times. In addition, error rates, total number of misses, response switching and response variability were derived from the task for each participant and time point. Response times and error rates were aggregated by taking the mean across trials at each time point, trial condition and block prior to further analysis. For response times, this aggregation took place after omitting incorrect responses and misses. For response times and error rates, data were additionally summarized as motor- (mean across choices) and selection-related (multiple choice > single choice) performance at each time point to facilitate correlation analyses. Patients responded with their most affected side. The responding hand of healthy controls was matched to the patients. To ensure that only participants who adhered to the task instructions were included for further analysis, time points with <25% correct one-choice trial responses averaged across blocks (indicating a randomized response pattern), or >60 missing responses (50% of choice trials) were excluded from further analysis.

### Image acquisition and processing

#### Scanning parameters

All MRI scans were acquired using a single Siemens MAGNETOM Prisma 3 T scanner (Siemens) equipped with a 32-channel head coil. Patients were scanned in the ON-medicated state. T1-weighted images were acquired using a magnetization-prepared rapid gradient-echo sequence [repetition time (TR)/echo time (TE)/inversion time (TI) = 2000/2/880 ms; flip angle = 8°; voxel size = 1.0 mm × 1.0 mm × 1.0 mm; slices = 192; field of view (FOV) = 256 mm; scanning time = 5 min]. Diffusion-weighted images were acquired using a multiband-accelerated echo-planar imaging sequence [TR/TE = 3600/92, flip angle = 90°, acceleration factor = 3, scheme = monopolar, b-values = 0–1000–2000/mm^2^, phase-encoding direction = anterior > posterior, voxel-size = 2.0 mm × 2.0 mm × 2.0 mm, slices = 72, FOV = 210, diffusion gradient directions = 104 (5 b0 images), scanning time = 6.5 min]. Field maps consisting of single b0 images with an inverted phase-encoding direction were acquired to correct for susceptibility distortions during subsequent image preprocessing. T2*-weighted images were acquired during the performance of the action selection task using a multi-band sequence (TR/TE = 1000/34 ms; acceleration factor = 6; acquisition mode = interleaved; flip angle = 60°; voxel-size = 2.0 mm × 2.0 mm × 2.0 mm; slices = 72; FOV = 210 mm; scanning time = 9–10 min).

#### Functional MRI processing

##### Preprocessing of functional images

FMRI data were preprocessed using fMRIPrep (v.23.0.2).^37^ Briefly, functional images were motion- and slice time-corrected before boundary-based registration to T1-weighted space^38^ and subsequent non-linear normalization to MNI152NLin6Asym-space (all interpolations were concatenated and applied in a single transformation).^39^ For participants with multiple time points, normalization to MNI-space was done via a participant-specific unbiased T1-weighted anatomical template.^40^ Lastly, images were smoothed with a Gaussian kernel of 6 mm at full-width half-maximum.

##### First-level analysis

Task-related activity was estimated with first-level analyses in SPM12 (https://www.fil.ion.ucl.ac.uk/spm/software/spm12), as described previously.^7^ Covariates for anatomical and motion-related sources of noise were included in each first-level model. Contrasts were set up to derive parameter estimates for two levels of choice (single choice > baseline; multiple choice > baseline), for motor-related activation (mean across choices > baseline) and for selection-related activation (multiple choice > single choice). Contrast images of left-sided responders were flipped horizontally, which ensured that the most-affected sides of patients were consistently on the right.

#### Structural MRI processing

##### Preprocessing of structural images

Diffusion-weighted MRI data were preprocessed using QSIPrep (v.0.19.0).^41^ Briefly, diffusion-weighted images were first subjected to Marchenko-Pastur principal component analysis denoising^42^ and Gibbs unringing,^43^ followed by corrections for head motion, eddy currents and susceptibility distortion.^44-47^ Lastly, images were resampled to 2 mm isotropic voxels in ACPC space. Diffusion tensor models were fitted to generate images of fractional anisotropy (FA), mean diffusivity (MD)^48,49^ and free water (FW).^50^ Additionally, average b0-images were computed. For each subject, an FA template was generated using mri_robust_register.^40^ Subject-specific FA templates from 50 healthy controls and 50 patients were combined to create a study-specific template using antsMultivariateTemplateConstruction, configured with the HCP165_FA_1mm template in MNI152NLin6Asym-space as the target.^39,51,52^

##### Substantia nigra free water

Degeneration of the posterior substantia nigra (pSN) was estimated in accordance with a well-established FW protocol previously used to investigate PD progression.^50,53^ Normalization of FW images followed previously established procedures: individual FW images were registered to subject-specific FA templates before being non-linearly normalized to the study-specific FA template.^54^ The same procedure was performed for b0 images, which were subsequently averaged and used to draw masks of the substantia nigra (SN; see the ‘Regions of interest’ section later).

##### Cortical surface-based mean diffusivity

Cortical degeneration was estimated as surface-based MD, a novel metric that has shown strong potential in monitoring progressive neurodegeneration,^55,56^ but which has not yet been applied to study PD. The FreeSurfer (v.7.3.2) longitudinal pipeline was used to generate cortical surfaces in T1-weighted space.^40^ Boundary-based registrations^38^ (6 degrees of freedom) were estimated from b0- to T1-weighted space and used to project MD onto cortical surfaces.^55,56^ Per vertex, this involved averaging MD values from six equidistant points between the white and pial surface, starting and stopping within 20% from the borders of each surface. Using this pipeline, surface-based images were created for uncorrected and FW-corrected MD.

### Regions of interest

#### Functional MRI

To test the main hypothesis of this study, a conservative region of interest (ROI) was generated based on results from a previously published baseline analysis of task-related activity that encompassed clusters relating to PD-related basal ganglia dysfunction and cortical compensation (Fig. 1C).^7^ Basal ganglia dysfunction was defined as regions showing reduced motor-related activity in patients compared with controls and included the bilateral putamen, left primary motor cortex and right cerebellar lobule IV–V. Cortical compensation was defined as regions showing an inverse correlation with bradykinesia severity and included a widespread network of parieto-premotor regions consisting of the bilateral superior parietal lobule, intraparietal sulcus, superior frontal gyrus and left middle frontal and paracingulate gyri. The ROI was combined with a horizontally flipped version to ensure equal coverage of both hemispheres. Maximum filtering was used to increase spatial coverage further and to merge smaller clusters into larger contiguous ones. The ROI was used for two purposes: to constrain voxel-wise group comparisons of longitudinal changes in task-related activity [i.e. investigations of the effect of time (baseline, follow-up)] and to constrain voxel-wise correlation analyses between longitudinal changes in task-related activity and clinical progression.

There were two exceptions where fMRI analyses were carried out using different ROIs from the main one defined above. First, to avoid circularity, group comparisons of basal ganglia activity at each separate time point, intended to test the reliability of PD-related dysfunction in the putamen, were performed using a whole-brain mask. Second, correlation analyses between selection-related activity and ON-state bradykinesia severity at each separate time point, intended to test the reliability of PD-related parieto-premotor cortex compensation, were performed on estimates that were extracted from an ROI composed of clusters showing an inverse relationship between selection-related activity and OFF-state bradykinesia severity at baseline.^7^

#### Structural MRI

The anterior SN and pSN were identified and drawn on an average b0 image, constructed from diffusion-weighted data of the same 50 healthy controls and 50 patients who contributed to the study-specific FA template (Fig. 1D).^53,54^ This procedure involved localizing the horizontal slice directly below the most inferior part of the red nucleus, where the SN appears hypointense. Masks of the anterior and pSN were drawn as 3 mm × 3 mm squares along three consecutive horizontal slices and subsequently used to extract FW values. The MD values were extracted from nine cortical regions of the Desikan–Killiany atlas^57^ (Fig. 1E) in subject-specific space.

### Statistical analysis

#### Longitudinal alterations in clinical severity, behavioural performance, brain activity and brain structure

Longitudinal changes in clinical severity and behavioural performance were investigated with linear mixed-effects modelling in R v.4.2.1 (R Core Team, 2022) using the lme4 package.^58^ Models were fitted using a restricted maximum likelihood approach, and *P*-values were derived from two-sided Wald χ^2^ tests in type III analyses of deviance. Age, sex and years of education were modelled as covariates of non-interest in all analyses. Disease duration (i.e. years since diagnosis) and dominance of the responding hand were added as additional covariates where appropriate. Dependent variables were log-transformed whenever possible.

In analyses of bradykinesia progression, time (baseline, follow-up) and medication (ON, OFF) were included as within-subjects factors. Repeated measurements were accounted for with by-subject random intercepts. Individual differences in progression rates were modelled with by-subject random slopes for time. Longitudinal changes in MoCA scores and LEDD were analysed in a similar manner after omitting the medication factor and by-subject random slopes.

In comparisons of behavioural performance between patients and controls, Group (control, patient) was included as a between-subjects factor. Time (baseline, follow-up) and Choice (single, multiple) were included as within-subjects factors. Trial block (one, two, three) was included as an additional within-subject factor to account for task habituation. The factors choice and trial block were omitted in analyses of button press variability, switching rates and misses. Linear mixed-effects models were used to analyse response times, button press variability and switching rates. Generalized linear mixed-effects models were used to analyse error rates (logistic regression) and misses (Poisson regression). The logistic mixed-effects model of error rates was weighted by number of trials to account for data aggregation. By-subject random intercepts accounted for repeated measurements, and by-subject random slopes for time were included when possible.

Group comparisons in brain activity were performed with AFNI's 3dLMEr function,^59,60^ closely following the analysis that was carried out for response times. Group (control, patient) was included as a between-subjects factor. Time (baseline, follow-up) and Choice (single, multiple) were included as within-subjects factors. By-subject random intercepts accounted for repeated measurements. By-subject random slopes of time were also included. Comparisons involving the effect of time were constrained to the ROI of basal ganglia dysfunction and cortical compensation (Fig. 1C). Reproducibility of previously observed effects for group and choice were investigated separately for each session, using a whole-brain mask to avoid circularity. Reproducibility of the relationship between bradykinesia severity and selection-related activity was assessed for each session by extracting the average beta estimate from a network showing this relationship at baseline.^7^ Cluster-forming thresholds were set at *P* < 0.001 (*Z* = 3.1).^61^ Multiple comparisons-corrected cluster extent thresholds were derived using 3dClustSim (10 000 iterations), based on the autocorrelation function of residuals estimated using 3dFWHMx, and considered significant at *P* < 0.05 (two-sided test).^62^ Anatomical labels were derived using the Anatomy Toolbox v.1.8,^63^ and Glasser^64^ atlases.

Group comparisons of pSN FW and cortical MD were subjected to similar linear mixed-effects modelling in R, omitting by-subject random slopes for time. For the pSN, an additional analysis was conducted within patients after adding hemisphere (contralateral to most- versus least-affected side of the body) as a within-subjects factor. The *P*-values in analyses of cortical MD were additionally adjusted for number of regions (*n* = 9) using the false discovery rate (FDR) method.

#### Associations with bradykinesia progression

Bradykinesia progression was correlated with longitudinal alterations in motor- and selection-related measurements of behavioural performance and brain activity and with measurements of brain structure (pSN FW and cortical MD) using multiple linear regression. To ensure that these associations related specifically to intrasubject variability and to account for regression to the mean effects, baseline scores for outcome and predictor variables were included as covariates of non-interest.^65^ Age, sex, years of education, disease duration and dominance of the responding hand were included as additional covariates. Years of education was included as a covariate and deliberate proxy for cognitive reserve, which can be defined as a premorbid accumulation of brain resources that delay the detrimental effects that neurodegeneration has on behaviour, to enable more specific inferences with respect to compensation.^7,24,31,66,67^ To enhance the specificity of inferences with respect to bradykinesia progression, all longitudinal correlation analyses were additionally adjusted for change in, and baseline scores of, cognitive performance (MoCA) and medication dosage (LEDD). The MoCA and LEDD scores were also added as covariates in all time-specific correlations of bradykinesia severity. For linear regressions between clinical and behavioural measurements, *P*-values were derived from *F*-tests in type III ANOVAs.

Four analyses were conducted with respect to bradykinesia progression in patients with complete data from both time points. First, the degree to which changes in ON- and OFF-state bradykinesia co-varied was explored. Second, change in motor- and selection-related behavioural performance was modelled as a function of bradykinesia progression. Third, associations were quantified between bradykinesia progression and brain activity change in a voxel-wise fashion using FSL's randomize function. Here, baseline brain activity was modelled as a voxel-wise covariate, and non-parametric permutation testing,^68^ combined with threshold-free cluster enhancement,^69^ was used to derive multiple comparisons corrected cluster extent thresholds. Fourth, associations with bradykinesia progression for pSN FW and cortical MD were quantified in the same way as for measurements of behavioural performance. The pSN FW was additionally related to motor symptom asymmetry.^10^ Analyses of bradykinesia progression accounted for longitudinal changes in cognitive performance and medication changes to enhance inference specificity. Control analyses were conducted to reproduce previous findings by correlating on-state bradykinesia severity with behavioural and brain measurements separately for baseline and follow-up.

## Results

### Clinical progression

#### Bradykinesia worsens over time, independently of medication status

**Figure 2:**
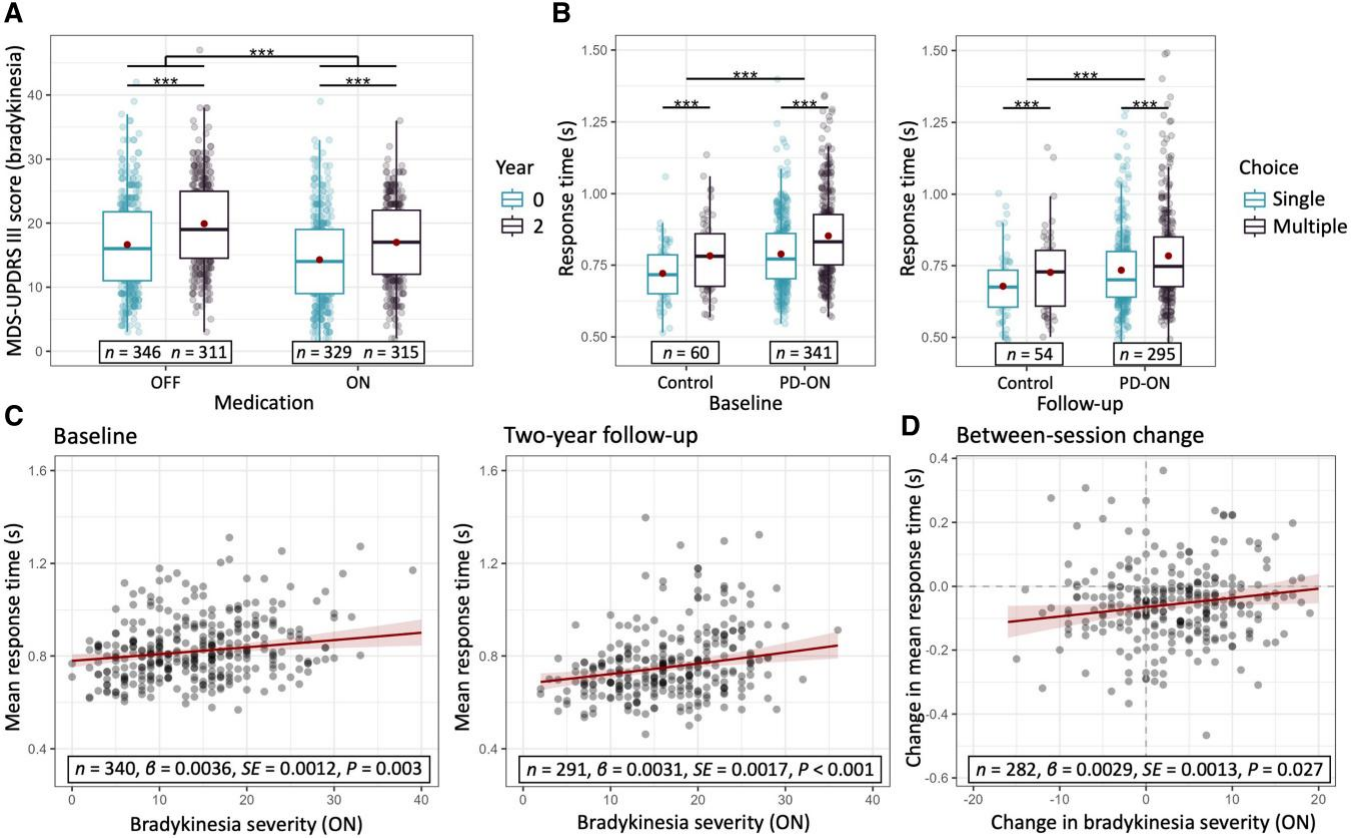
**Clinical progression and longitudinal alterations in behavioural performance.** (**A**) Two-year bradykinesia progression. (**B**) Response times in the action selection task. (**C**) Correlation between bradykinesia progression and longitudinal change in mean response times. (**D**) Correlations between ON-state bradykinesia severity and mean response times at baseline (*left*) and follow-up (*right*). ***P < 0.001. MDS-UPDRS = Movement Disorder Society Unified Parkinson's Disease Rating Scale; SE = standard error of the mean.

Bradykinesia increased by approximately three points over 2 years [Fig. 2A; time effect: χ^2^(1) = 84.01, *P* < 0.001, $\eta_{p}^{2}$ = 0.21; follow-up > baseline: β = 3.32, SE = 0.36, *t-*ratio(321) = 9.17, *P* < 0.001], and it decreased following medication [medication effect: χ^2^(1) = 282.00, *P* < 0.001, $\eta_{p}^{2}$ = 0.31; on > off: β = −2.78, SE = 0.17, *t*-ratio(636) = 16.79, *P* < 0.001]. There were no differences in bradykinesia progression between ON- and OFF-medicated states (Time × Medication effect: *P* = 0.14). Bradykinesia progression in ON- and OFF-medicated states was correlated substantially [Supplementary Fig. 1; *F*(1,280) = 395.83, *P* < 0.001, $\eta_{p}^{2}$ = 0.64, β = 0.86, SE = 0.04]. Given the strong correspondence between OFF- and ON-state clinical scores, only the latter was used for subsequent analyses.

#### Bradykinesia progression co-varies with cognitive decline and medication titration

Over time, cognitive performance declined [Time effect: χ^2^(1) = 54.26, *P* < 0.001, $\eta_{p}^{2}$ = 0.15; follow-up > baseline: β = −0.41 (equivalent to a 1.12 point decline in non-adjusted MoCA scores), SE = 0.06, *t*-ratio(301) = 7.37, *P* < 0.001], whereas medication dosages increased [Time effect: χ^2^(1) = 165.40, *P* < 0.001, $\eta_{p}^{2}$ = 0.35; follow-up > baseline: β = 217.03, SE = 16.88, *t*-ratio(306) = 12.86, *P* < 0.001]. Furthermore, longitudinal decline in cognitive performance was correlated with bradykinesia progression [*F*(1,279) = 13.80, *P* < 0.001, $\eta_{p}^{2}$ = 0.002, β = −0.03, SE = 0.01], although increasing medication dosages was not (*P* = 0.45). To enhance the specificity of associations involving bradykinesia, cognitive decline and medication titration were added as covariates of non-interest in subsequent correlation analyses.

### Longitudinal analyses of behavioural performance

#### Parkinson's disease impairs overall motor performance

Patients responded more slowly compared with controls [Fig. 2B; Group effect: χ^2^(1) = 11.18, *P* < 0.001, $\eta_{p}^{2}$ = 0.03; patient > control: log-ratio = 1.07, SE = 0.02, *t*-ratio(370) = 3.34, *P* < 0.001]. Additionally, all participants responded slower as demands on action selection increased, although this was less pronounced at follow-up compared with baseline [Time × Choice: χ^2^(1) = 4.67, *P* = 0.031, $\eta_{p}^{2}$ = 0.001; follow-up > baseline, multiple > single: log-ratio = 0.99, SE = 0.006, *t*-ratio(4941) = 2.16, *P* = 0.031]. There was no indication that PD patients became slower over time compared with healthy controls (linear mixed-effects model, Group × Time and Group × Time × Choice effects: both *P* > 0.69).

Analyses of additional behavioural metrics (error rates, number of misses and response flexibility) showed that PD patients and healthy controls performed the action selection task using similar strategies (Supplementary material).

#### Behavioural performance indexes bradykinesia progression and severity

Faster bradykinesia progression was associated with longitudinal slowing of motor-related response times [Fig. 2C; *F*(1,269) = 4.94, *P* = 0.027, β = 2.893 × 10^−3^, SE = 1.302 × 10^−3^]. Additionally, motor-related response times increased as a function of ON-state bradykinesia severity at both baseline [Fig. 2D; *F*(1,331) = 9.24, *P* = 0.003, β = 3.609 × 10^−3^, SE = 1.187 × 10^−3^] and follow-up [*F*(1,282) = 12.81, *P* < 0.001, β = 6.046 × 10^−3^, SE = 1.690 × 10^−3^]. Error rates and number of misses correlated similarly with bradykinesia severity (Supplementary material).

### Longitudinal analyses of task-related brain activity

#### Parkinson's disease impairs movement-related processing in the basal ganglia

**Figure 3:**
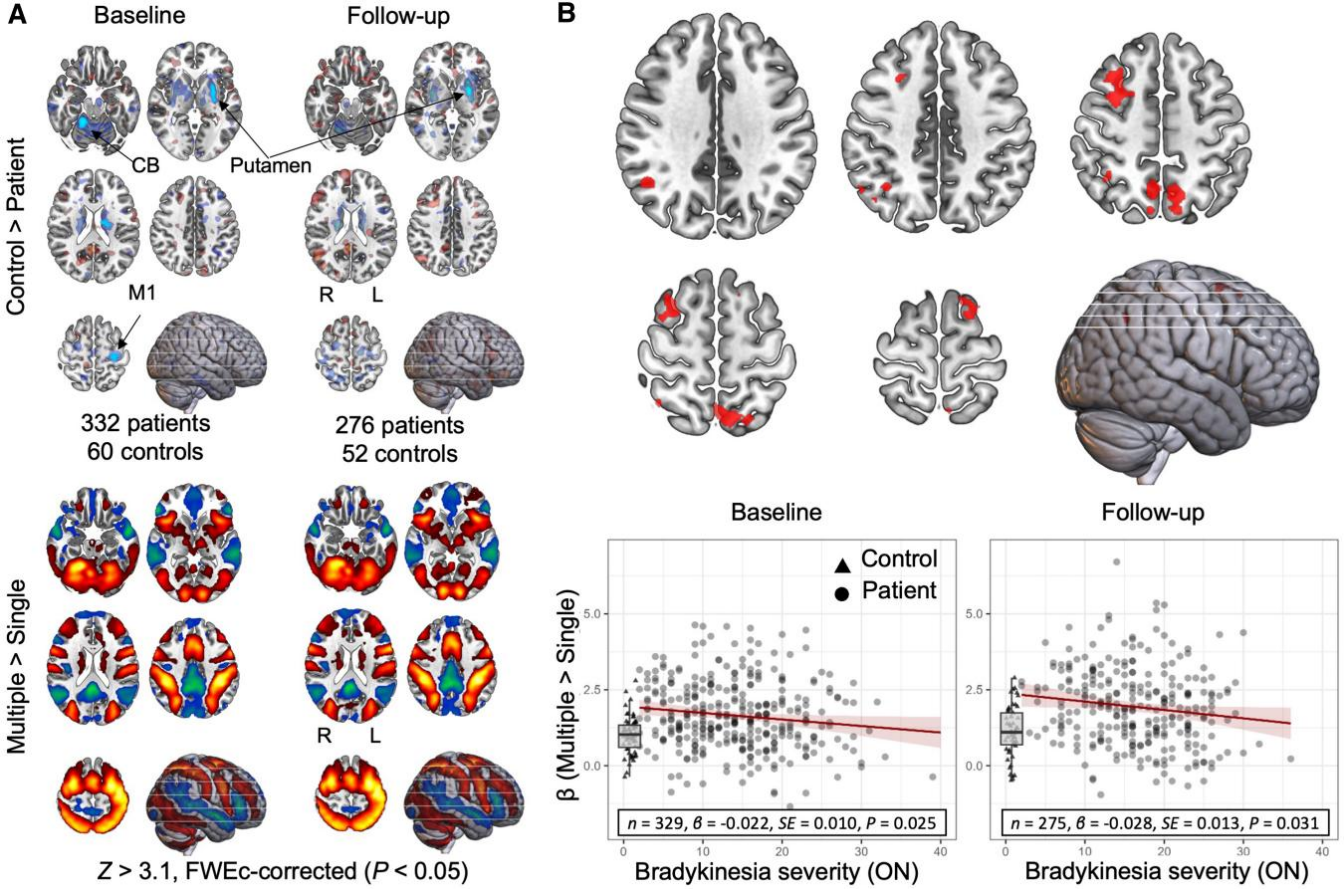
**Repeatability of task-related effects and brain-clinical correlations.** (**A**) Reduced movement-related putamen activity in patients compared with controls (*top*) and the effect of increasing action selection demand (*bottom*) is present at both baseline and 2-year follow-up. (**B**) Correlations between selection-related activity and off-state bradykinesia severity observed at baseline in a previous publication (*top*) is also present in the on-state at baseline (*bottom left*) and at follow-up (*bottom right*). Box plots display the range of selection-related activity for healthy controls. CB = cerebellum; FWEc = familywise error-corrected, cluster-level; L = left; M1 = primary motor cortex; R = right; SE = standard error of the mean.

PD patients showed reduced motor-related activity in the putamen compared with healthy controls (Fig. 3A, top and Table 2). This effect was reliably present at a conservative whole-brain level at both baseline and at follow-up. Additionally, increasing action selection demand reliably elicited strong parieto-prefrontal network activation and default-mode network deactivation for both PD patients and healthy controls (Fig. 3A, bottom). However, analogous to the analysis of response times, there were no differences in longitudinal change of selection-related activity between PD patients and healthy controls.

**Table 2:** Voxel-wise mixed-effects comparisons of longitudinal changes in brain activity between patients and healthy controls

Anatomical label (% cluster volume in area)	Area	*P*-value (FWEc-corrected)	Cluster extent (voxels)	FWEc-corrected cluster extent threshold (*α* = 0.05; *P* < 0.001; voxels)^a^	Max *z*-value	MNI: *x*, *y*, *z*
**Group: Control > Patient (across sessions)** ^b^						
L putamen (82%)	–	*P* < 0.0001	650	36	7.65	−26, −2, 3
R putamen (67%)	–	*P* < 0.0001	183	36	5.75	28, −6, 0
R cerebellum (88%)	IV–V	*P* < 0.0001	135	36	4.84	16, −50, −20
L precentral gyrus (94%)	M1	*P* < 0.02	51	36	4.07	−28, −24, 63
**Group: Patient > Control (across sessions)** ^b^						
NS	–	–	–	36	–	–
**Group: Control > Patient (baseline)** ^b^						
L putamen (82%)	–	*P* < 0.0001	470	36	7.26	−27, −4, 3
R cerebellum (92%)	IV–V	*P* < 0.0001	171	36	6.23	16, −48, −20
L precentral gyrus (92%)	M1	*P* < 0.0001	131	36	4.61	−29, −25, 62
R putamen (96%)	–	*P* < 0.02	52	36	5.14	30, −5, 4
**Group: Patient > Controls (baseline)** ^b^						
NS	–	–	–	36	–	–
**Group: Control > Patient (follow-up)**						
L putamen (91%)	–	*P* < 0.0001	189	36	4.74	−26, −1, 2
R putamen (54%)	–	*P* < 0.03	46	36	4.58	28, −9, 1
**Group: Patient > Controls (follow-up)**						
NS	–	–	–	36	–	–
**Group × Time**						
NS	–	–	–	36	–	–
**Group × Time × Choice**						
NS	–	–	–	36	–	–

FWEc = familywise error, cluster-corrected; L = Left; MNI = Montreal Neurological Institute; NS = non-significant, *P* > 0.05; R = Right.

^a^Simulated based on the autocorrelation function of residuals. Anatomical labels and areas are derived from the Anatomy Toolbox v1.8 (CA_ML_18_MNI) and Glasser (MNI_Glasser_HCP_v1.0) atlases, respectively.

^b^Effects derived using a whole-brain mask instead of the region of interest in Fig. 1C, which was partly based on findings at baseline.

#### Bradykinesia progression is determined by longitudinal changes in cortical compensation

**Figure 4:**
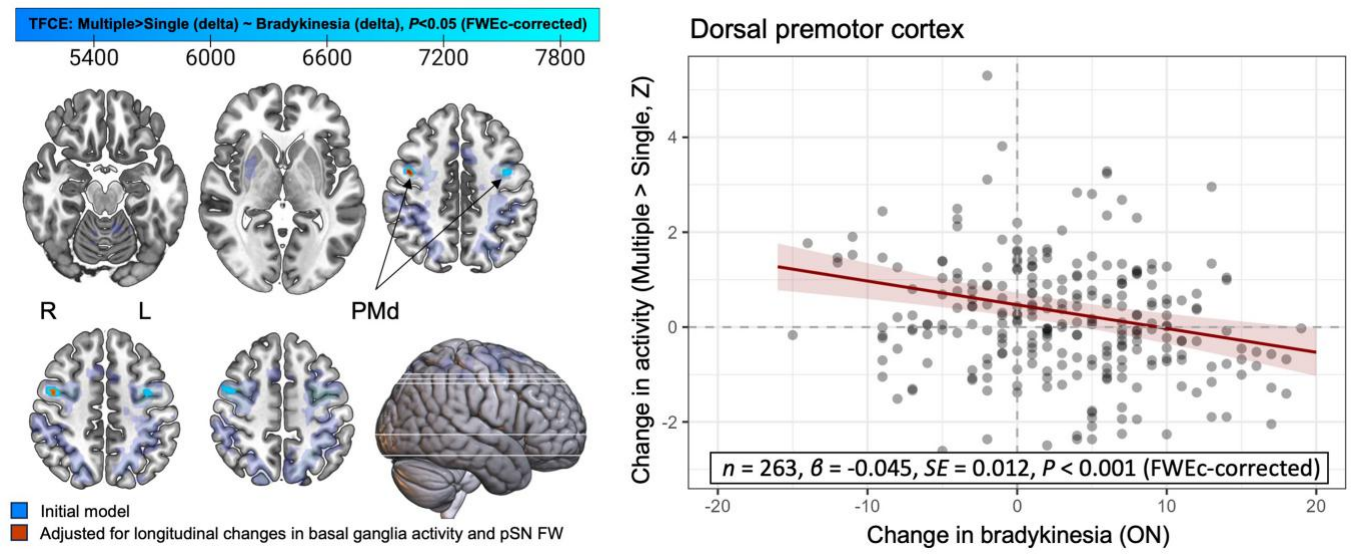
**Longitudinal correlation between bradykinesia and selection-related premotor cortex activity.** Two-year bradykinesia progression is inversely correlated with longitudinal changes in selection-related activity across the extent of the region of interest (blue), reaching the threshold for statistical significance only in the bilateral dorsal premotor cortex. After adjusting for longitudinal changes in the functional and structural integrity of the nigrostriatal system, the cluster in the right hemisphere remains significant (red). FW = free water; FWEc = familywise error, cluster-level; PMd = premotor cortex, dorsal; pSN = posterior substantia nigra; SE = standard error of the mean; TFCE = threshold-free cluster enhancement.

In line with the main hypothesis, bradykinesia progression was negatively correlated with longitudinal change in selection-related activity in the right and left dorsal premotor cortex (Fig. 4 and Table 3). These correlations were similarly present for bradykinesia progression assessed in the OFF-medicated state (Supplementary material, Supplementary Table 1 and Supplementary Fig. 2). To control for potential contributions of nigrostriatal deficits, the analysis above was repeated after including longitudinal changes in selection-related putamen activity and pSN FW as additional covariates of non-interest. The previously identified cluster in the right premotor cortex remained significant, whereas the relatively weaker cluster in the left premotor cortex did not (Fig. 4 and Table 3). There were no additional correlations between longitudinal changes in motor/selection-related activity and cognitive performance or medication titration, nor with sex or education (all *P* > 0.1).

**Table 3:** Voxel-wise correlation analyses between bradykinesia progression and longitudinal changes in brain activity

Anatomical label (% cluster volume in area)	Area	*P*-value (FWEc-corrected)	Cluster extent (voxels)	Maximum TFCE	MNI: *x*, *y*, *z*
**ΔMultiple > Single (selection-related activity) ∼ ΔBradykinesia**					
**Positive correlation**					
NS	–	–	–	–	–
**Negative correlation**					
R precentral gyrus (99%)	FEF	0.005	97	8007	45, −3, 44
L precentral gyrus (86%)	FEF	0.03	46	5207	−44, −3, 42
**ΔMultiple > Single (selection-related activity) ∼ ΔBradykinesia, adjusted for nigrostriatal dysfunction**					
**Positive correlation**					
NS	–	–	–	–	–
**Negative correlation**					
R precentral gyrus (100%)	FEF	0.022	28	5956	43, −3, 44
**ΔMean > Baseline (motor-related activity) ∼ ΔBradykinesia**					
**Positive correlation**					
NS	–	–	–	–	–
**Negative correlation**					
NS	–	–	–	–	

FWEc = familywise error, cluster-corrected; L = Left; MNI = Montreal Neurological Institute; NS = non-significant, *P* > 0.05; R = Right; TFCE = threshold-free cluster enhancement. Anatomical labels and areas are derived from the Anatomy Toolbox v1.8 (CA_ML_18_MNI) and Glasser (MNI_Glasser_HCP_v1.0) atlases, respectively.

#### Selection-related activity reliably indexes bradykinesia severity

In an ROI analysis based on previous cross-sectional findings (Fig. 3B, top),^7^ selection-related activity decreased as a function of ON-state bradykinesia severity at both baseline [Fig. 3B, bottom left; *F*(1,321) = 5.05, *P* = 0.025, $\eta_{p}^{2}$ = 0.02] and at follow-up [Fig. 3B, bottom right; *F*(1,267) = 4.68, *P* = 0.031, $\eta_{p}^{2}$ = 0.034].

### Longitudinal analyses of brain structure

#### Parkinson's disease-specific degeneration of the posterior substantia nigra

**Figure 5:**
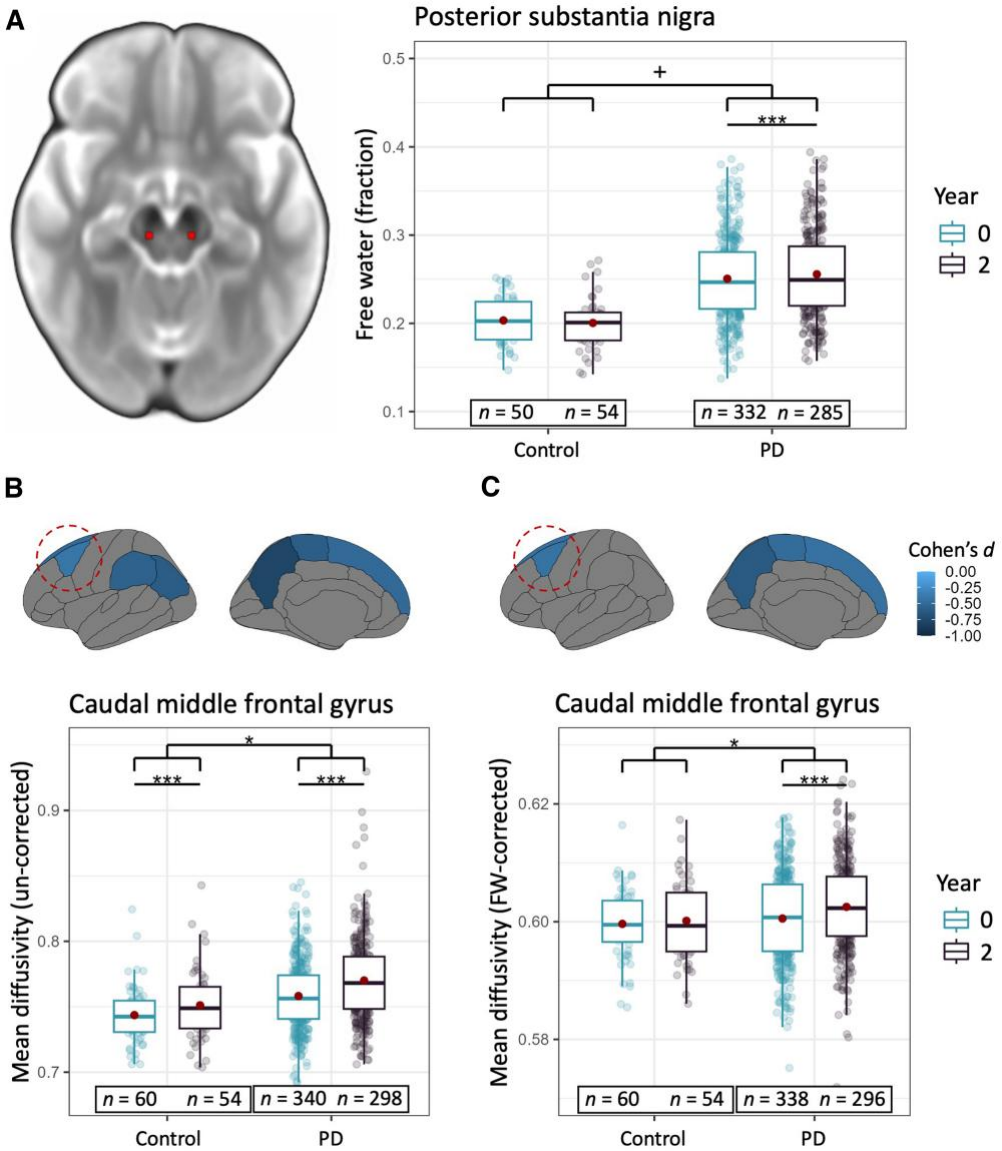
**Effects of PD on longitudinal alterations in brain structure.** (**A** and **B**) PD is associated with increased pSN FW (**A**), and with increased MD in caudal middle frontal, superior frontal, inferior parietal, supramarginal, precuneus, and paracentral cortex (**B**). (**C**) After correcting for FW, increased MD is confined to the caudal middle frontal, paracentral, precuneus, supramarginal and superior frontal cortex. ^+^*P* = 0.086, *P < 0.05, ***P < 0.001. FW = free water; MD = mean diffusivity; PD = Parkinson’s disease; pSN = posterior substantia nigra.

There was a trend towards a larger increase in pSN FW in PD patients compared with healthy controls [Fig. 5A and Supplementary Table 2; Group × Time effect: χ^2^(1) = 2.95, *P* = 0.086]. *Post hoc* analyses of longitudinal FW changes in each group revealed a clear increase in PD patients [follow-up > baseline: log-ratio = 1.027, SE = 0.008, *t*-ratio(340) = 3.35, *P* < 0.001], but not in healthy controls (*P* = 0.60).

The analysis above was repeated within PD patients after identifying the sides of the SN that were contralateral to the clinically most- and least-affected sides of the body. As before, there was an increase in pSN FW over time [Time effect: χ^2^(1) = 13.17, *P* < 0.001; follow-up > baseline: log-ratio = 1.048, SE = 0.008, *t*-ratio(319) = 3.63, *P* = <0.001]. Additionally, FW was higher in the pSN contralateral to the clinically most-affected side of the body [Hemisphere effect: χ^2^(1) = 11.38, *P* < 0.001, most > least: log-ratio = 1.048, SE = 0.015, *t*-ratio(563) = 3.37, *P* < 0.001]. There were no differences in longitudinal change between hemispheres (Time × Hemisphere effect: *P* = 0.82).

#### Posterior substantia nigra degeneration indexes motor symptom asymmetry, but not clinical progression

There was no correlation between bradykinesia progression and longitudinal changes in pSN FW (Supplementary Table 3; *P* = 0.62), nor between pSN FW and bradykinesia severity at either baseline (Supplementary Table 4) or follow-up (Supplementary Table 5).

Right–left asymmetry of motor symptoms was correlated negatively with right–left asymmetry in pSN FW at both baseline [*F*(1,323) = 9.49, *P* = 0.002, β = −0.068, SE = 0.022] and follow-up [*F*(1267) = 8.60, *P* = 0.004, β = −0.092, SE = 0.031]. However, there was no relationship between longitudinal change in the two variables (*P* = 0.64).

#### Parkinson's disease-specific structural changes in the cerebral cortex

In six of nine cortical ROIs, PD patients showed greater longitudinal increases in cortical MD compared with controls (Fig. 5B and Supplementary Table 2). This included the caudal middle frontal cortex [Group × Time effect: χ^2^(1) = 5.54, *P*_FDR_ = 0.028, $\eta_{p}^{2}$ = 0.015] and superior frontal cortex [Group × Time effect: χ^2^(1) = 8.36, *P*_FDR_ = 0.007, $\eta_{p}^{2}$ = 0.023], where bradykinesia progression was correlated with selection-related activity.

#### Cortical structure does not index clinical progression in Parkinson's disease

There was no correlation between longitudinal changes in cortical MD and bradykinesia progression (Supplementary Table 3). At baseline, bradykinesia severity was correlated with MD in the inferior parietal, precuneus, superior frontal and supramarginal cortices (Supplementary Table 4). At follow-up, there were no correlations with bradykinesia severity (Supplementary Table 5).

To enhance the specificity of MD with respect to microstructural damage, a *post hoc* analysis of FW-corrected cortical MD was done.^55^ Here, longitudinal PD-related increases in FW-corrected cortical MD compared with healthy controls were confined to four of nine cortical ROIs (Fig. 5C and Supplementary Table 2), including the caudal middle frontal cortex [Group × Time effect: χ^2^(1) = 6.72, *P*_FDR_ = 0.025, $\eta_{p}^{2}$ = 0.019] and the superior frontal cortex [Group × Time effect: *χ*^2^(1) = 6.44, *P*_FDR_ = 0.025, $\eta_{p}^{2}$ = 0.018]. As previously, there were no correlations between FW-corrected cortical MD and bradykinesia progression (Supplementary Table 3). Moreover, FW-corrected cortical MD was not correlated with bradykinesia severity at either baseline (Supplementary Table 4) or follow-up (Supplementary Table 5).

#### Longitudinal changes in task-related premotor cortex activity are not associated with localized changes in structure

A *post hoc* analysis was done to test whether loss of cortical compensation might have resulted from progressive microstructural damage. Selection-related premotor cortex activity was not correlated with surface-based MD (all *P* > 0.5) or FW-corrected MD (all *P* > 0.1) in either the middle or the superior frontal cortex.

## Discussion

We tested the hypothesis that progressive worsening of motor symptoms in PD over 2 years is associated with gradual loss of compensatory function in the cerebral cortex (quantified with task-based fMRI), over and above nigrostriatal degeneration (quantified with FW imaging of the pSN).^7^ There are two main sets of findings. First, we provide empirical evidence for our hypothesis that (hyper)activity in the parieto-premotor cortex serves a compensatory role and that the degree of cortical compensation shapes the clinical course of PD. Specifically, in line with our previous cross-sectional work, we demonstrate that bradykinesia severity is negatively correlated with selection-related activity in the parieto-premotor cortex at two separate time points, further supporting the view that enhanced cortical function compensates for underlying deficits in the nigrostriatal motor network.^7^ In addition, we show that individual rates of bradykinesia progression are correlated specifically with longitudinal changes in selection-related activity in the premotor cortex, even when accounting for the functional and structural integrity of the nigrostriatal system. Second, we provide empirical evidence for the canonical finding that nigrostriatal cell loss in PD leads to severe striatal dysfunction.^8-10^ Specifically, PD patients showed increased levels of pSN FW (a measure of nigral cell damage) in comparison to healthy controls, which progressed over 2 years and was more pronounced in the most- versus least-affected hemisphere. Furthermore, PD patients showed consistent reductions in motor-related activity in the putamen across two time points. However, neither of these measures was correlated with bradykinesia severity or progression. Taken together, our findings indicate that the progression of bradykinesia is shaped by the loss of cortical compensation, and not only by deficits in the nigrostriatal system.

### The role of the nigrostriatal system in progression of PD

Despite the prominent role assigned to nigrostriatal deficits in producing the symptoms of PD,^10^ we observed no relationships between structural or functional indices of nigrostriatal integrity and clinical symptom progression. In PD patients compared with healthy controls, our imaging analyses revealed decreased putamen activity, which remained stable over 2 years, and elevated FW in the pSN, which increased over time. These results are consistent with previous findings from both cross-sectional and longitudinal MRI studies of PD,^8,9,53,70^ which supports the soundness of our methodological approach.

The previously observed lack of relationships between dopamine depletion and clinical progression might reflect how pathology propagates through the nigrostriatal system. It has been argued that the highly branched dopaminergic (and noradrenergic) neurotransmitter systems undergo a ‘dying-back’ process of degeneration in PD, such that striatal dopamine terminals perish first, followed by loss of cell bodies in the SN pars compacta.^71-75^ Our findings are consistent with this idea; deficits in motor-related activity in the putamen of PD patients remained unchanged over the course of 2 years, whereas the reduced structural integrity of the pSN deteriorated further. However, it should be emphasized that structural and fMRI approaches might not be directly comparable when monitoring progression owing to differences in sensitivity. Additionally, it should be noted that we measured task-based activity while patients were on their usual dopaminergic treatment, which was continuously titrated from baseline to follow-up. Increasing medication dosages might have led to normalization of putamen activity, thereby explaining the lack of progression. Arguing against this, we have previously shown, using an ON-versus-OFF design, that dopaminergic medication did not influence putamen activity elicited by the action selection task.^7^ Furthermore, inclusion of dopaminergic medication dose as a covariate did not change any of the findings.

Although our task was designed specifically to assess motor deficits of the clinically most-affected side, recent evidence indicates that clinical progression in early PD might be more strongly related to dopaminergic decline in the putamen contralateral to the least-affected side.^23^ This is likely to be because dopaminergic denervation on the least-affected side is relatively less advanced, reflecting a difference in the dynamics of the mechanisms contributing to the symptom severity of each side. It is conceivable that a longitudinal relationship between clinical progression and putamen activity could have been observed if our task had also targeted the least-affected side.

Lastly, we would like to emphasize that our findings by no means suggest that nigrostriatal deficits are clinically irrelevant for PD progression; the most-affected side of the pSN reliably reflected the clinically most-affected side of the body, as did motor-related activity reductions in the putamen. Moreover, the level of asymmetric degeneration in the pSN was proportional to the asymmetric distribution of motor symptoms. This indicates that nigrostriatal deficits are a necessary condition for the development of motor symptoms (and explains why dopamine replacement therapy remains an effective treatment for alleviating motor disability), but probably without being the primary source of their progressive worsening in the years following the diagnosis.

### The role of cortical compensation in Parkinson's disease progression

In line with our main hypothesis, we found that the progressive worsening of motor symptoms was correlated with a progressive decline in dorsal premotor hyperactivity, which we argue is compensatory in nature.^7^ Hyperactivity in the cerebral cortex has been observed consistently in neuroimaging studies of PD,^8,9^ particularly in response to cognitively demanding tasks,^76^ and has often been interpreted as a compensatory phenomenon.^26^ However, hyperactivity might also reflect maladaptive changes, such as loss of inhibition, that exacerbate symptoms and contribute to disease progression.^77^ For instance, although increased cortical input to the striatum might reflect a compensatory adaptation in early stages of PD, it could potentially also hasten the degeneration of nigrostriatal terminals owing to increased energy demands.^75,78^ The inverse correlation between premotor cortex activity and symptom severity, both cross-sectionally at each time point and when assessing longitudinal changes, suggests that the premotor activity we observed here reflects a compensatory mechanism. A contributing factor might be that the dorsal premotor cortex is functionally connected to anterior territories of the striatum,^79^ which remain relatively spared from pathology until later disease stages.^25,80^ Furthermore, parietal and premotor areas are among the last cortical regions to atrophy owing to synuclein pathology, potentially enabling them to retain their capacity for compensatory adjustments into later disease stages, when their functionality starts to degrade owing to spreading neurodegeneration, without becoming maladaptive.^29,81^

To understand how cortical compensation can be preserved, it is essential to identify underlying processes that contribute to its decline. To this end, we used diffusion-based imaging to measure microstructural damage longitudinally in cortical grey matter.^55,56^ We demonstrated that PD leads to an accelerated structural decline in several cortical regions, including the premotor cortex. However, structural cortical changes were not correlated with the speed of clinical progression or with changes in task-related activity. More sensitive methods might be required to identify relationships between decline in cortical compensation and structure.

Our finding that longitudinal changes in task-related premotor cortex activity relate to the speed at which bradykinesia worsens opens new opportunities to design progression-slowing interventions. During movement planning, activity in the primary motor cortex is biased towards contextually appropriate motor plans by input from a network of premotor and parietal areas.^82^ Enhancing these biasing influences might enable the premotor cortex to compensate more effectively for deficits in basal ganglia output. Interestingly, studies in healthy individuals have demonstrated that stimulation of the parieto-frontal cortex can alter the excitability of the primary motor cortex,^83-85^ which might translate into beneficial effects on motor performance in PD.^86,87^ Recently, it has been shown that the primary motor cortex of the clinically least-affected hemisphere compensates for loss of dopamine through enhanced plasticity and reduced interhemispheric inhibition, potentially making it more receptive to additional compensatory input from upstream cortical areas, such as the premotor cortex.^88,89^ Further research is needed to ascertain how stimulation of the parieto-premotor cortex alters compensatory influences over primary motor cortex activity and whether such influences can be leveraged to slow down clinical progression.

### Interpretational issues

The relationship we observed between individual differences in the speed of clinical progression and compensatory collapse needs to be viewed in the light of results from our group-level comparisons. Although symptoms progressively worsened across PD patients, there was no consistent reduction in compensation over time, arguing against the possibility that its relationship with clinical progression was mediated by ageing-related influences, which occurred similarly for both patients and controls. More importantly, the lack of group-level effects on compensation strongly implies that PD patients follow their own unique trajectories of compensatory collapse, rather than a strictly linear decline. These trajectories might reflect underlying heterogeneity in PD-related pathology, giving rise to subtypes characterized by opposing directions of longitudinal change that contribute to clinical progression despite cancelling out effects at a group level.^7,34,90,91^ This heterogeneity is apparent in Fig. 3B, where the between-subject variability of selection-related activity is markedly more pronounced for PD patients than it is for healthy controls. Detecting individual differences in trajectories of compensatory collapse might be key to identifying patients at risk of rapid progression.

Our approach to quantifying nigrostriatal deficits has limitations. First, task-based activity in the putamen might lack the sensitivity required to detect subtle longitudinal changes in comparison to nuclear imaging.^22^ Second, our method for quantifying pSN degeneration is relatively coarse. Alternative imaging modalities, such as neuromelanin-sensitive MRI or quantitative susceptibility imaging, combined with automated segmentation of functional territories, might yield better sensitivity to disease progression.^92-95^

It could be argued that scanning in an ON-medicated state might have led to a loss in sensitivity with respect to detecting associations with ‘pure’ disease progression. However, even after medication withdrawal, there are long-lasting effects of dopaminergic medication that provide symptomatic relief lasting for days.^96^ Moreover, understanding the mechanisms underlying disease progression in an ON-medicated state holds intrinsic value because it more closely reflects the everyday life of patients.

## Conclusion

We provide empirical evidence supporting the hypothesis that clinical progression in PD is associated with a collapse of compensatory function in the dorsal premotor cortex. Further research is now required to demonstrate whether delaying this collapse can have disease-modifying effects. Emerging evidence already indicates that compensation can be preserved through lifestyle alterations that promote neuronal health, such as physical activity,^6,97,98^ and through targeted neuromodulation techniques that temporarily boost cortical function.^87^ In combination, the complex neurophysiological effects that these non-pharmacological interventions evoke might have the potential to both elicit and retain compensatory enhancements, thereby contributing to the attenuation of clinical progression in PD beyond what is currently achievable through more traditional pharmacological treatments.

## Supplementary Material

awaf302_Supplementary_Data

## Data Availability

Data supporting the findings of this study are available upon request only, to ensure the privacy of participants. A data acquisition request can be sent to the corresponding author or using the following website: https://www.personalizedparkinsonproject.com/data–sharing/requesting. Analysis code is openly available at https://github.com/mejoh/PPP_imaging.
