# Supplementary material for: Parkinson's disease progression is shaped by longitudinal changes in cerebral compensation
Source: Brain. 2025 Aug 12;149(1):194–208. doi: 10.1093/brain/awaf302 (PMC12782169; doi:10.1093/brain/awaf302)
Supplement: awaf302_Supplementary_Data [file awaf302_supplementary_data.pdf]

Supplementary material

Missingness of data

At baseline, 351 patients and 60 controls participated. 329 patients and 56 controls returned for two-year follow-up measurements.

Clinical data

At baseline, 23 ON-state (*N*=329) and 6 OFF-state (*N*=346) patients lacked clinical data. At two-year follow-up, 14 ON-state (*N*=315) and 18 OFF-state (*N*=311) patients lacked clinical data.

Task performance

At baseline, 10 patients lacked behavioral task data (*N*=341; 4 missing data; 7 poor performance). At two-year follow-up, 34 patients (*N*=291; 24 missing data; 10 poor performance) and 2 controls (*N*=54; 2 missing data) lacked behavioral task data.

Functional MRI

At baseline, 30 patients lacked functional MRI data (*N*=332; 1 missing scan; 4 missing behavioral data; 13 technical issues; 3 poor performance; 9 poor image quality). At two-year follow-up, 53 patients (*N*=276; 28 missing scan; 10 missing behavioral data; 3 technical issues; 3 poor performance; 9 poor image quality) and 4 controls (*N*=52; 2 missing scan; 2 quality control) lacked functional MRI data.

Diffusion-weighted MRI

At baseline, 1 patient lacked diffusion-weighted MRI data (*N*=351; 1 missing scan). At two-year follow-up, 22 patients (*N*=307; 22 missing scan) and 2 controls (*N*=54; 2 missing scan) lacked diffusion-weighted data.

For analyses of substantia nigra free water, 19 patients were excluded at baseline (*N*=332; 7 technical issues, 12 perivascular spaces) and 22 patients were excluded at follow-up (*N*=285; 10 technical issues, 12 perivascular spaces). Additionally, 10 controls were excluded at baseline (*N*=50) and at follow-up (*N*=45) due to perivascular spaces.

For analyses of cortical mean diffusivity, 13 patients were excluded at baseline (*N*=338; 13 technical issues) and 11 patients were excluded at follow-up (*N*=296; 11 technical issues).

Behavioral performance metrics pertaining to response strategies

PD-related behavioral deficits may prompt patients to adopt response strategies in the action selection task that differ from the response strategies of healthy controls. This may introduce additional variability into measurements of brain activity, thereby exerting confounding influences on our functional MRI analyses. To ascertain whether such a confounding influence may be present in our data, we investigated additional metrics of behavioral performance that more closely capture potential discrepancies in response strategies between PD patients and healthy controls. In addition, we examined whether changes in these behavioral performance metrics correlated with bradykinesia progression.

Error rates

PD leads to cognitive deficits and impaired coordination of individual finger movements, which may impair action selection capabilities. In the action selection task, this may manifest as increased error rates, resulting from responses that do not reflect intended targets. We therefore investigated whether PD differentially influenced longitudinal changes in error rates. We observed a general increase in error rates (logistic mixed-effects model, TIME effect: $\chi^2(1)=8.75$, $P=0.003$; follow-up>baseline: $OR=1.56$, $SE=0.23$), suggestive of an aging-related decline in accuracy. Alternatively, in light of our finding that response times decreased over time, this may indicate that participants favored speed over accuracy to a greater extent at follow-up compared to baseline. We also observed a trend towards higher error rates in PD patients compared to healthy controls (logistic mixed-effects model, GROUP effect: $\chi^2(1)=3.29$, $P=0.07$; patient>control: $OR=1.43$, $SE=0.28$), and a trend towards a general increase in error rates for multiple-choice compared to single-choice (logistic mixed-effects model, CHOICE effect $\chi^2(1)=3.05$, $P=0.08$; multiple>single: $OR=1.24$, $SE=0.15$).

We observed no relationship between longitudinal changes in error rates and bradykinesia progression (logistic mixed-effects model: $P=0.47$). Nevertheless, we observed that error rates correlated with the severity of bradykinesia. More specifically, bradykinesia severity correlated with motor-related error rates (multiple linear regression: $F(1,334)=13.22$, $P<0.001$, $\beta=1.41\text{e-}03$, $SE=3.90\text{e-}04$) and selection-related error rates (multiple linear regression: $F(1,334)=9.218$, $P=0.003$, $\beta=-1.20\text{e-}03$, $SE=4.0\text{e-}04$) at baseline. There were no correlations between error rates and bradykinesia at follow-up. These findings suggest that appropriate target selection may become more difficult as bradykinesia severity increases (multiple linear regression: $P>0.13$).

Missed responses

PD also impairs the ability to initiate actions, potentially resulting in more missed responses in the action selection task. We therefore investigated whether PD differentially influenced longitudinal changes in numbers of missed responses. We observed a longitudinal increase in misses, which was smaller for patients than controls (Poisson mixed-effects model, GROUP×TIME effect: $\chi^2(1)=5.56$, $P=0.018$; follow-up>baseline, patients>controls: $OR=0.59$, $SE=0.13$), although both groups missed more responses over time (follow-up>baseline, patients: $OR=1.33$, $SE=0.08$, *z-ratio*=4.62, $P<0.001$; follow-up>baseline, controls: $OR=2.26$, $SE=0.49$, *z-ratio*=3.77, $P<0.001$). Consistent with our analysis of error rates, these results point to aging-related effects on cognition,

demonstrating that PD has limited effects on the ability to respond accurately and timely to the action selection task.

We observed no association between bradykinesia progression and longitudinal change in misses (multiple linear regression: P=0.68). Nevertheless, we observed that number of misses correlated with bradykinesia severity. More specifically, bradykinesia severity correlated with number of misses at both baseline (Poisson regression: $\chi^2(1)$=44.00, P<0.001, IRR=1.04, SE=0.006) and follow-up (Poisson regression: $\chi^2(1)$=51.45, P<0.001, IRR=1.05, SE=0.008).

Response flexibility

PD places constraints on the ability to flexibly adjust response strategies according to changes in the environment, manifesting as a tendency to repeat responses rather than selecting new ones. In this study, participants were asked to vary their responses as much as possible during the action selection task, enabling us to test whether PD is characterized by reduced behavioral flexibility, and how this reduced flexibility may relate to clinical progression, which we investigated in two ways.

Response switching

First, we examined whether PD patients were more prone to repeating responses in trials where they had the option to switch to a new one. We quantified response switching ability as the ratio between numbers of response repeats and response switches, accounting only for trials in which both repeats and switches were simultaneously possible. We then tested whether PD differentially influenced longitudinal changes in response switching. We observed a trend towards lower switching rates in PD patients compared to healthy controls (linear mixed-effects model, GROUP effect: $\chi^2(1)$=3.69, P=0.055; patient>control: *log-ratio*=0.90, SE=0.05). These findings indicate that PD patients may be more prone to repetitive responding strategies.

We also observed that faster bradykinesia progression was associated with longitudinal decreases in switching rates (multiple linear regression: $F(1,277)$=4.38, P=0.037, β=-0.41, SE=0.19), suggesting that responding strategies become more repetitive as symptoms worsen. However, there were no correlations between bradykinesia at either baseline (multiple linear regression: P>0.9) or follow-up (multiple linear regression: P>0.18).

Response variability

Second, we examined whether PD patients had reduced variability in selecting responses throughout the action selection task. We quantified response variability as the coefficient of variation among the four possible response options. We then investigated whether PD differentially influenced longitudinal changes in response variability. We observed that PD patients had higher response variability compared to healthy controls (linear mixed-effects model, GROUP effect: $\chi^2(1)$=4.49, P=0.034, η^2_p=0.012; patient>control: *log-ratio*=1.12, SE=0.10, *t-ratio*(380)=2.12, P=0.035). We also observed that response variability generally increased over time (linear mixed-effects model, TIME effect: $\chi^2(1)$=4.60, P=0.032, η^2_p=0.013; follow-up>baseline: *log-ratio*=1.13, SE=0.06, *t-ratio*(355)=2.14, P=0.033). These findings indicate that PD patients were capable of varying their responses during the action selection task. This

eliminates the potential confound that PD patients and healthy controls differ in their reliance on habitual versus goal-directed action selection during the task.

We observed no association between bradykinesia progression and longitudinal change in response variability (multiple linear regression: P=0.37). Nevertheless, we observed that bradykinesia severity correlated with response variability at baseline (multiple linear regression: $F(1,334)=5.12$, P=0.024, β=3.18e-03, SE=1.41e-03), but not at follow-up (multiple linear regression: P=0.39).

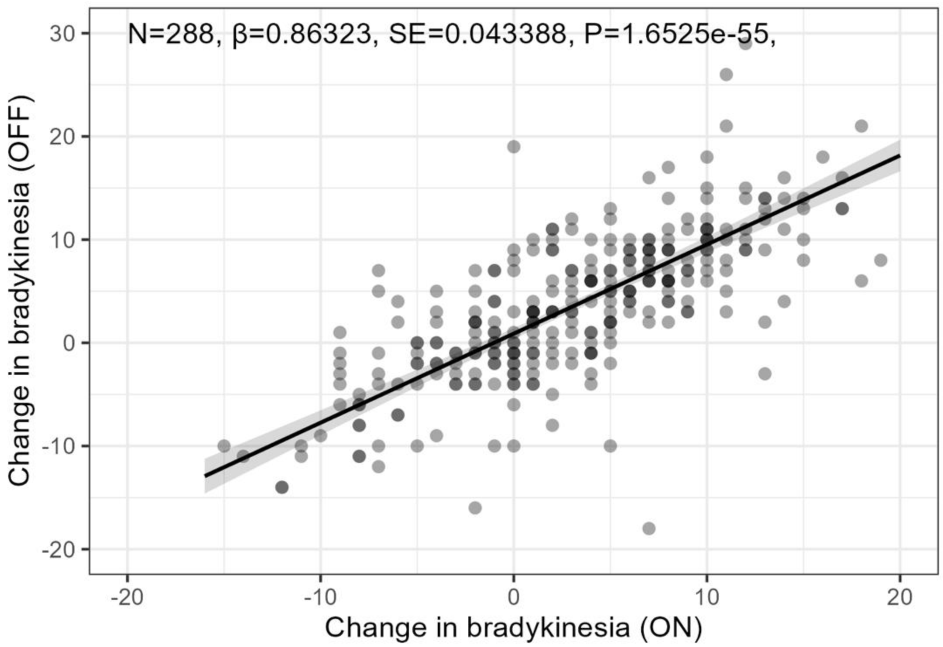

Supplementary Figure 1. Correlation between bradykinesia progression assessed in ON- and OFF-medicated states.

Longitudinal changes in cortical compensation correlate with off-state assessment of bradykinesia progression

Bradykinesia progression, assessed in the OFF-medicated state, showed significant negative correlations with longitudinal changes in selection-related activity in the left precentral gyrus, right middle frontal gyrus, right precentral gyrus, left postcentral gyrus, and left inferior parietal lobule (**Supplementary Table 1**). However, these correlations did not remain significant after accounting for longitudinal changes in selection-related putamen activity and posterior substantia nigra free water.

Supplementary Table 1 Voxel-wise correlation analyses between bradykinesia progression and longitudinal changes in brain activity

Anatomical label (% cluster volume in area)	Area	P-value (FWEc-corrected)	Cluster extent (voxels)	Max TFCE	MNI: X, Y, Z
ΔMultiple > Single (selection-related activity) ~ ΔBradykinesia					
**Positive correlation**					
Ns.					
**Negative correlation**					
L precentral gyrus (53%)	6d	0.021	406	4126	-25,-7,68
R middle frontal gyrus (99%)	I6-8	0.043	22	3645	41,1,60
L precentral gyrus (95%)	6d	0.045	22	3335	-27,-21,72
R precentral gyrus (55%)	6d	0.045	14	3639	27,-13,70
L postcentral gyrus (100%)	2	0.048	9	3586	-33,-33,50
L inferior parietal lobule (100%)	AIP	0.047	7	3234	-42,-41,46
ΔMultiple > Single (selection-related activity) ~ ΔBradykinesia, adjusted for nigro-striatal dysfunction					
**Positive correlation**					
Ns.					
**Negative correlation**					
Ns.					
ΔMean > Baseline (motor-related activity) ~ ΔBradykinesia					
**Positive correlation**					
Ns.					
**Negative correlation**					
Ns.					

Anatomical labels and areas are derived from the Anatomy Toolbox v1.8 (CA_ML_18_MNI) and Glasser (MNI_Glasser_HCP_v1.0) atlases, respectively.

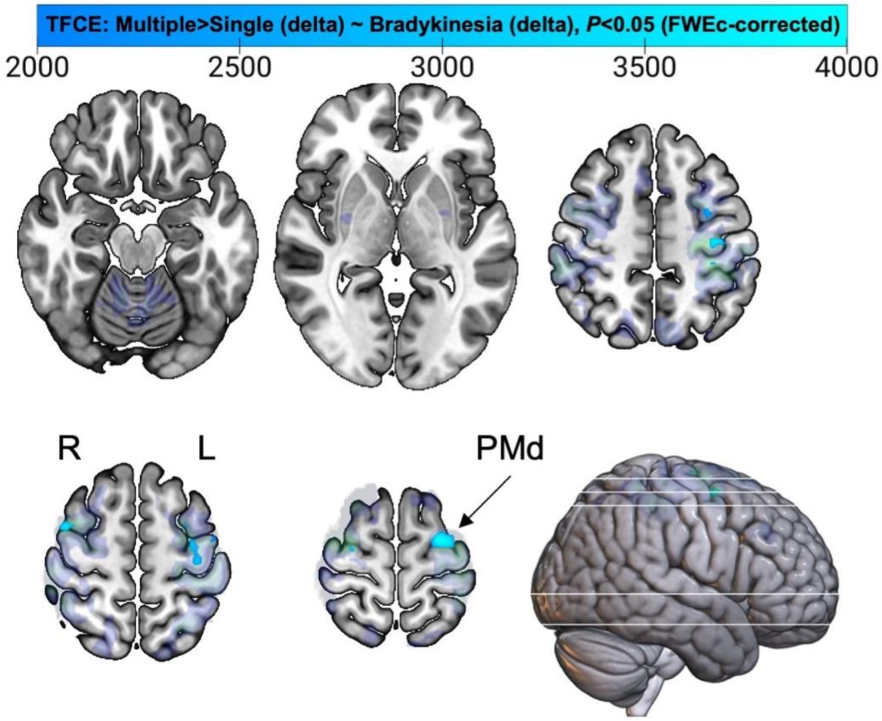

Supplementary Figure 2. Longitudinal correlation between OFF-state bradykinesia and selection-related activity. Two-year bradykinesia progression, assessed OFF dopaminergic medication, correlates inversely with longitudinal changes in selection-related activity, reaching significance only in bilateral dorsal premotor cortex and left inferior parietal lobule (blue). FWEc=Familywise error, cluster-level; PMd=Premotor cortex, dorsal; TFCE=Threshold-free cluster enhancement.

Supplementary Table 2 Group analyses of longitudinal changes in brain structure.

Area	Healthy	PD	GROUP×TIME (χ^2)	Effect size (η^2_p)
Δ*Substantia nigra free water*				
Anterior	0.998 (0.042)	0.973 (0.016)	0.332	-
Posterior	0.990 (0.020)	1.027 (0.008)***	2.946+	0.031
Δ*Cortical mean diffusivity (un-corrected)*				
Caudal middle frontal	1.009 (0.003)***	1.016 (0.001)***	5.523*	0.015
Inferior parietal	1.009 (0.003)***	1.020 (0.001)***	14.377***	0.039
Paracentral	1.006 (0.004)	1.022 (0.002)***	12.867**	0.035
Postcentral	1.011 (0.003)***	1.017 (0.001)***	3.596	-
Precentral	1.015 (0.003)***	1.015 (0.001)***	0.025	-
Precuneus	1.002 (0.003)	1.017 (0.001)***	22.650***	0.060
Superior frontal	1.008 (0.002)***	1.016 (0.001)***	8.357**	0.023
Superior parietal	1.015 (0.003)***	1.019 (0.001)***	1.748	-
Supramarginal	1.010 (0.003)***	1.020 (0.001)***	11.067**	0.031
Δ*Cortical mean diffusivity (free water-corrected)*				
Caudal middle frontal	1.000 (0.001)	1.003 (0.001)***	6.717*	0.019
Inferior parietal	1.000 (0.002)	1.001 (0.001)+	0.219	-
Paracentral	1.001 (0.001)	1.005 (0.001)***	9.332*	0.026
Postcentral	1.005 (0.001)***	1.003 (0.001)***	1.156	-
Precentral	1.005 (0.001)***	1.003 (0.001)***	1.689	-
Precuneus	0.998 (0.001)+	1.002 (0.000)***	16.046***	0.044
Superior frontal	1.001 (0.001)	1.004 (0.000)***	6.443*	0.018
Superior parietal	1.003 (0.001)**	1.003 (0.000)***	0.186	-
Supramarginal	1.002 (0.001)	1.003 (0.001)***	0.155	-

Values for each group are displayed as log-ratios (standard errors) of differences between sessions (follow-up – baseline) extracted from linear mixed-effects models using the *emmeans* package in R. P-values for GROUP×TIME interactions in cortical MD analyses were adjusted for multiple comparisons using the false-discovery rate method (n=9). Δ=Follow-up>Baseline, +=P<0.1, *=P<0.05, **=P<0.01, ***=P<0.001.

Supplementary table 3 Correlations between bradykinesia progression and longitudinal changes in structural MRI-based metrics.

Area	F-statistic	P-value	Estimate (β)	Standard error of estimate
∆Bradykinesia ~ ∆Substantia nigra free water				
Anterior	0.629	0.428	5.979	7.536
Posterior	0.247	0.620	-5.058	10.178
∆Bradykinesia ~ ∆Cortical mean diffusivity (un-corrected)				
Caudal middle frontal	0.655	0.419	-1.584e-04	1.957e-04
Inferior parietal	2.015	0.157	-2.619e-04	1.845e-04
Paracentral	0.756	0.385	-2.565e-04	2.949e-04
Postcentral	1.522	0.219	-2.827e-04	2.292e-04
Precentral	1.140	0.287	-2.576e-04	2.413e-04
Precuneus	3.366	0.068	-3.839e-04	2.093e-04
Superior frontal	0.249	0.875	-2.729e-05	1.728e-04
Superior parietal	4.738	0.030	-4.391e-04	2.017e-04
Supramarginal	1.040	0.309	-2.236e-04	2.193e-04
∆Bradykinesia ~ ∆Cortical mean diffusivity (free water-corrected)				
Caudal middle frontal	0.327	0.568	-3.218e-05	5.625-e05
Inferior parietal	2.511	0.114	2.014e-04	1.271e-04
Paracentral	0.003	0.954	3.996e-05	6.851e-05
Postcentral	0.311	0.578	3.914e-05	7.018-e05
Precentral	0.250	0.617	-3.389e-05	6.775e-05
Precuneus	0.569	0.451	-3.609e-05	4.785e-05
Superior frontal	0.349	0.556	3.591e-05	6.078e-05
Superior parietal	0.135	0.714	3.459e-05	9.423e-05
Supramarginal	3.746	0.054	1.394e-04	7.205e-05

P-values from cortical mean diffusivity analyses were adjusted for multiple comparisons using the false-discovery rate method (n=9). +=P<0.1, *=P<0.05, **=P<0.01, ***=P<0.001.

Supplementary table 4 Correlations between bradykinesia severity and structural MRI-based metrics at baseline.

Area	F-statistic	P-value	Estimate (β)	Standard error of estimate
Bradykinesia severity ~ Substantia nigra free water				
Anterior	2.837	0.093	3.543e-03	2.103e-03
Posterior	2.659	0.104	-2.576e-03	1.580e-03
Bradykinesia severity ~ Cortical mean diffusivity (un-corrected)				
Caudal middle frontal	5.207	0.0232	5.772e-04	2.530e-04
Inferior parietal	6.985	8.635e-03*	7.659e-04	2.898e-04
Paracentral	1.001	0.318	3.255e-04	3.253e-04
Postcentral	3.147	0.077	5.863e-04	3.305e-04
Precentral	2.661	0.104	5.178e-04	3.174e-04
Precuneus	4.278	0.0394*	5.916e-04	2.860e-04
Superior frontal	5.358	0.0212*	5.226e-04	2.258e-04
Superior parietal	0.576	0.449	2.741e-04	3.612e-04
Supramarginal	7.708	5.832e-03*	7.711e-04	2.778e-04
Bradykinesia severity ~ Cortical mean diffusivity (free water-corrected)				
Caudal middle frontal	1.129	0.289	1.108e-04	1.043e-04
Inferior parietal	1.288	0.257	1.116e-04	9.831e-05
Paracentral	0.495	0.482	8.744e-05	1.243e-04
Postcentral	0.832	0.362	1.053e-04	1.154e-04
Precentral	1.155	0.283	1.389e-04	1.292e-04
Precuneus	2.771	0.097	1.331e-04	7.997e-05
Superior frontal	1.600	0.207	1.096e-04	8.662e-05
Superior parietal	0.889	0.347	9.986e-05	1.059e-04
Supramarginal	1.608	0.258	1.169e-04	9.218-e05

P-values from cortical mean diffusivity analyses were adjusted for multiple comparisons using the false-discovery rate method (n=9). +=P<0.1, *=P<0.05, **=P<0.01, ***=P<0.001.

Supplementary table 5 Correlations between bradykinesia severity and structural MRI-based metrics at follow-up.

Area	F-statistic	P-value	Estimate (β)	Standard error of estimate
Bradykinesia severity ~ Substantia nigra free water				
Anterior	0.054	0.816	-6.313e-04	2.718e-03
Posterior	0.267	0.606	9.847e-04	1.907e-03
Bradykinesia severity ~ Cortical mean diffusivity (un-corrected)				
Caudal middle frontal	0.242	0.623	1.747e-04	3.548e-04
Inferior parietal	1.159	0.283	4.202e-04	3.903e-04
Paracentral	0.623	0.431	3.541e-04	4.488e-04
Postcentral	0.214	0.644	1.973e-04	4.267e-04
Precentral	0.583	0.446	3.108e-04	4.072e-04
Precuneus	0.017	0.896	5.038e-04	3.869e-04
Superior frontal	1.178	0.279	3.307e-04	3.048e-04
Superior parietal	0.486	0.486	-3.116e-04	4.469e-04
Supramarginal	2.109	0.148	5.267e-04	3.627e-04
Bradykinesia severity ~ Cortical mean diffusivity (free water-corrected)				
Caudal middle frontal	0.011	0.918	1.328e-04	1.287e-04
Inferior parietal	4.890	0.0291	3.221e-04	1.472e-04
Paracentral	1.492	0.223	1.968e-04	1.611e-04
Postcentral	0.826	0.364	1.448e-04	1.593e-04
Precentral	0.395	0.530	1.016e-04	1.617e-04
Precuneus	0.268	0.605	5.764e-05	1.114e-04
Superior frontal	0.785	0.376	1.001e-04	1.130e-04
Superior parietal	0.148	0.700	5.268e-05	1.368e-04
Supramarginal	6.028	0.015	2.966e-04	1.208e-04

P-values from cortical mean diffusivity analyses were adjusted for multiple comparisons using the false-discovery rate method (n=9). +=P<0.1, *=P<0.05, **=P<0.01, ***=P<0.001.
